# Comparison of in-patient glucose team based management with conventional blood glucose management- a retrospective study from China

**DOI:** 10.1186/s13098-023-01242-3

**Published:** 2024-01-03

**Authors:** Jiayu Lin, Jinying Zhang, Bo Liang, Jinkuang Lin, Neng Wang, Jialin Lin, Huibin Huang

**Affiliations:** 1 Department of Endocrinology, The Second Affiliated Hospital of Fujian Medical University, Quanzhou, Fujian 362000 China; 2 Department of Neurology, The Second Affiliated Hospital of Fujian Medical University, Quanzhou, Fujian 362000 China; 3 Department of Medical administration, The Second Affiliated Hospital of Fujian Medical University, Quanzhou, Fujian 362000 China; 4 Department of Information, The Second Affiliated Hospital of Fujian Medical University, Quanzhou, Fujian 362000 China

**Keywords:** Diabetes, Inpatient glucose team management, Hospital days, Health-care costs, Nomogram

## Abstract

**Background:**

Glycemic control for patients with diabetes in the surgical department is often unsatisfactory. Compounding this issue is the fact that conventional glucose management models are often inefficient and difficult to monitor over time.

**Objective:**

To investigate the impact of inpatient glucose team-based management on glycemic control and hospital days in surgical patients with diabetes.

**Methods:**

A retrospective analysis was conducted on 4156 patients with diabetes in the surgical department who received inpatient management of diabetes at a tertiary medical center from June 2020 to May 2022. Based on whether they received inpatient glucose team-based management, the surgical patients with diabetes were divided into two groups: the inpatient glucose team-based management (GM group, consisting of 1698 participants) and the conventional blood glucose management group (control group, consisting of 2458 participants). We compared the two groups in terms of glycemic control, hospital days, and health-care costs. Multiple logistic regression analysis was performed to build the hospital days prediction model and nomogram. Finally, the performance of the model was evaluated.

**Results:**

The rate of glucose detection was higher in the GM group at 2 h postprandial (P < 0.01). The incidence of hypoglycemia and severe hyperglycemia, blood glucose attainment time, pre-operative preparation days, hospital days, and health-care costs were lower in the GM group than in the control group (P < 0.01). The linear regression model revealed that blood glucose attainment time, incidence of hypoglycemia (< 3.9mmol/L), preoperative preparation days, perioperative complications, and health-care costs were the factors influencing the hospital days (Total Point 83.4 points, mean hospital days 9.37 days). Receiver operating characteristic (ROC) curve analysis demonstrated that the nomogram had good accuracy for predicting hospital days (area under the ROC curve 0.83, 95% confidence interval [CI], 0.74 to 0.92).

**Conclusion:**

Inpatient glucose team-based management demonstrated significant improvements in glycemic control among surgical patients with diabetes, resulting in reduced hospital days and associated costs. The developed nomogram also exhibited promising potential in predicting hospital days, offering valuable clinical applications.

## Introduction

Hyperglycemia and hypoglycemia are common occurrences among hospitalized patients and can result in adverse outcomes. Hyperglycemia affects millions of patients annually, increasing the risk of both infectious and noninfectious complications [1, 2], mortality rates [3, 4], hospital days, and costs [5]. With approximately one-quarter of hospitalized patients diagnosed with diabetes, inpatient hyperglycemia is a significant issue. The prevalence of hyperglycemia ranges from 32 to 38%, with higher rates observed in surgical patients [6, 7]. Hypoglycemia also poses a threat to hospitalized patients, as it increases the likelihood of both mortality and complications [8, 9].

Previously, patients with diabetes in the surgical department were managed independently through endocrine consultation guidance for hyperglycemia. However, several issues such as insufficient timeliness, irregular and inaccurate blood glucose monitoring, and lack of professional knowledge and skills among surgical medical staff have made it difficult to achieve standard blood glucose control. Numerous studies have demonstrated that hyperglycemia is an independent risk factor for postoperative infection, poor wound healing, increased incidence of postoperative complications, ICU admissions, and even mortality [10]. Additionally, hospitalized patients may experience impaired organ function, such as liver impairment and renal insufficiency, which limit the use of hypoglycemic drugs. Patients may also require inter-departmental transfers during hospitalization, such as transfers from intensive care units to general wards, which necessitate coordinated management and consultation from multiple departments. This process can give rise to conflicts in areas such as blood glucose monitoring, interrupted medication application, and inadequate internal communication among healthcare professionals, all of which are obstacles to achieving optimal glucose management [11].

Inpatient glucose team-based management involves establishing a hospital-wide glucose management team with the endocrinology department at its core. Through standardized management practices, this team formulates individualized glucose control goals, glucose-lowering programs, and follow-up plans for the entire hospital, particularly for perioperative hyperglycemic patients [12]. Inpatient glucose team-based management modalities can reduce adverse events resulting from glucose metabolism disorders and achieve objectives such as reducing pre-operative preparation days, shortening hospital days, and lowering health-care costs [13]. With the development of information technology, the trend towards informationization of inpatient blood glucose management has emerged. Our hospital piloted in-surgery glucose management for a range of specialties (spine surgery, arthrosurgery, urological surgery, neurosurgery, hepatobiliary surgery, thoracic surgery, gastrointestinal surgery, ophthalmology, otorhinolaryngology, and gynecology) by an inpatient glucose management team. This study explores the effectiveness and safety of an inpatient glucose team-based management model for patients with diabetes in the surgical department.

## Materials and methods

### Subjects and group classification

We conducted a study at the Second Hospital of Fujian Medical University to compare the effectiveness of inpatient glucose management (GM) model versus the conventional blood glucose management methods for non-critical surgery patients with diabetes. Since June 2021, our hospital implemented inpatient blood glucose management for patients with diabetes in the surgical department. Based on whether they received inpatient glucose team-based management, the surgical patients with diabetes were divided into two groups: the inpatient glucose team-based management (GM group) and the conventional blood glucose management group (control group). The GM group included 1698 participants admitted to our hospital from June 2021 to May 2022. This group consisted of 1261 elective surgeries (74.24%) and 437 urgent surgeries (25.76%). The control group consisted of 2458 participants admitted between June 2020 and May 2021, with 1859 elective surgeries (75.63%) and 599 urgent surgeries (24.37%). Although COVID-19 was characterized as a pandemic by the WHO as of March 11, 2020, the COVID-19 pandemic occurred in our region from December 2022 to January 2023 and there were no recorded cases of COVID-19 infection in our hospital’s vicinity during our study period. Therefore, the dates did not coincide with our study period. This study was reviewed and authorized by the Ethics Committee at the Second Hospital of Fujian Medical University, and it adhered to the principles of the Declaration of Helsinki. The ethics approval number for this study is Lun Audit (Research) No. 234 of 2021.

Inclusion criteria were as follows: (1) patients who were hospitalized for more than 24 h and were at least 18 years old, (2) diabetes diagnosis criteria [14]: symptoms of diabetes plus plasma glucose ≥ 11.1 mmol/L at any time, or fasting plasma glucose ≥ 7.0 mmol/L, or glycated hemoglobin > = 6.5%, or plasma glucose ≥ 11.1 mmol/L at 2 h of OGTT. Exclusion criteria were as follows: (1) stress hyperglycemia: patients without a history of diabetes, but with hospital hyperglycemia > 140 mg/dL, (2) patients with poor compliance or mental and neurological disorders that prevented them from cooperating, (3) pregnant or lactating women, (4) patients with severe multi-organ failure, (5) patients with uncontrollable infection, defined as patients who continue to experience ongoing infection symptoms, abnormal laboratory indicators, positive bacterial cultures, abnormal imaging findings, or clinical judgment indicating ineffective disease control despite appropriate treatment measures. (6) patients transferred to the intensive care unit during hospitalization.

The routine methods of point-of-care glucose measurement for both groups included the following, for those with greater glucose fluctuations, monitoring of blood glucose 7 times (including monitoring of fasting glucose, preprandial glucose, 2-hour postprandial glucose, and bedtime glucose). For those with smaller fluctuating blood glucose, blood glucose was monitored 5 times (including monitoring of fasting glucose, 2-hour postprandial glucose, and bedtime glucose).

About inpatient treatment of hyperglycemia: (1) For patients on an insulin therapy regimen, a basal-bolus subcutaneous insulin therapy regimen was used, based on meals and sleep times. If the patient was receiving continuous nutrition, short- or rapid-acting insulin was administered every 4–6 h. For patients with poor dietary intake, the basal-plus regimen was performed. Insulin pump therapy was also an option if conditions permit. (2) For patients with a stable clinical conditions and regular eating habits, outpatient treatment for diabetes was maintained, using the same oral hypoglycemic drugs or GLP-1 receptor agonists that were in use before hospitalization.

### Group and intervention

#### GM group

The inpatient glucose management team is comprised of several key members, including an endocrinology physician, an endocrinology nurse, a specialist liaison from another department, as well as representatives from the nutrition, information, and pharmacy departments. Endocrinology doctors utilize the virtual ward module within the Hitech system to monitor blood glucose levels of patients across various departments. They proactively manage the glucose of patients who do not meet the standard, making timely adjustments to their glucose-lowering plan based on changes in their condition and glucose levels. Additionally, nurses provide one-on-one bedside education tailored to each patient’s unique condition. They also follow up with patients on a daily basis to ensure proper glucose management.

The operation process for inpatient glucose management involves several key steps. Firstly, patients with diabetes in the surgical department who have poor glycemic control will have their inpatient glucose management application submitted by the managing physician via the electronic medical record system. Next, the application is reviewed by the endocrinologist and included in the virtual ward of the program. Appropriate glucose control goals are set for the patients and dietary and exercise programs are developed. Health education is also conducted to ensure patients understand their glucose management plan. Daily glucose management is then carried out by the endocrinologist and endocrinology nurses. The endocrinologist checks the blood glucose monitoring records daily and adjusts the glycemic lowering plan as needed, while working closely with the nutrition department to formulate an appropriate diet plan. Endocrinology nurses are responsible for disassembling and installing insulin pumps, setting basal rates, and providing patient education on pump loading. Upon discharge, the endocrinology physician adjusts the glucose-lowering program and prescribes diabetes medication in a timely manner after receiving the discharge prompt. Nurses in the endocrinology department are responsible for the recovery of insulin pumps, insulin injectors, and other equipment and provide educational materials for follow-up after discharge. The nurse in charge of the patient’s department instructs patients on discharge medication, including proper insulin injection technique and how to take diabetes-related drugs.

#### Control group

A departmental management or traditional counseling model for glucose management was used, and nurses manually recorded glucose data. If the surgeon felt that the patient’s blood glucose was not up to standard (before a meal ≥ 7.8 mmol/L, after a meal ≥ 10.0 mmol/L, blood glucose ≤ 3.9 mmol/L), the surgeon would send a consultation request to the endocrinology department. The endocrinology consultant physician received the consultation request and wrote a consultation opinion, but did not follow up on the implementation and the patient’s glucose levels. Surgeons will adjust a patient’s glucose-lowering plan based on consultation.

### Data collection and outcome measures

The following indicators were recorded for both groups: (1) Basic indicators: gender, age, duration of diabetes, fasting blood glucose (FBG), glycated hemoglobin (HbA1c), and 2-hour postprandial blood glucose. (2) Glycemic control indexes: incidence of severe hyperglycemia (blood glucose > 16.7 mmol/L), incidence of hypoglycemia (blood glucose ≤ 3.9 mmol/L), incidence of clinically significant hypoglycemia (blood glucose < 2.2 mmol/L), and blood glucose attainment time. (3) Other management effectiveness indicators: days of preoperative preparation, the incidence of perioperative complications (Nosocomial infection), days of hospitalization, and health-care costs (RMB).

For elective surgeries (major, intermediate, minor), fasting blood glucose should be controlled between 6.1 and 7.8 mmol/L. Postprandial (2 h after a meal) or random blood glucose should be controlled between 7.8 and 10.0 mmol/L. For urgent surgeries (major, intermediate, minor), fasting blood glucose should be controlled between 7.8 and 10.0 mmol/L. Postprandial or random blood glucose should be controlled between 7.8 and 13.9 mmol/L. For delicate surgeries, fasting blood glucose should be controlled between 4.4 and 6.1 mmol/L. Postprandial or random blood glucose should be controlled between 6.1 and 7.8 mmol/L [15]. Delicate surgeries refer to surgical procedures that require delicate and precise manipulation of organs or tissues, such as plastic surgery.

A hypoglycemic event was defined as any recorded data showing a blood glucose level ≤ 3.9 mmol/L. Hypoglycemia was classified into three categories: Grade 1 < 70 mg/dL (3.9 mmol/L), Grade 2 < 54 mg/dL (3.0 mmol/L)), and Grade 3 < 40 mg/dL (2.2 mmol/L) [16]. Severe hypoglycemic events were defined as any recorded data indicating a glycemic level of ≤ 2.2 mmol/L. Severe hyperglycemic events were defined as any recorded data indicating a glycemic level of > 16.7 mmol/L. Hypoglycemia incidence was calculated as the percentage of hypoglycemic events to the total number of patients with blood glucose monitoring, multiplied by 100%. Similarly, severe hypoglycemia incidence was calculated as the percentage of severe hypoglycemic events to the total number of patients with blood glucose monitoring, multiplied by 100%. Incidence of severe hyperglycemia was calculated as the percentage of severe hyperglycemic events to the total number of patients with blood glucose monitoring, multiplied by 100%.

### Statistical analyses

Analyses were performed in R (version 4.2.2) for all statistical analyses. The continuous variables were expressed as mean ± standard deviation and categorical variables were expressed as n (%). The Student’s t-test or one-way ANOVA test was used for continuous variables when appropriate. The Chi-square test or Fisher’s exact test was used for categorical variables when appropriate. Associated coefficients and predictive power were assessed using Spearman’s correlation analysis and ROC analysis, respectively. Figures were plotted using the “ggplot2”, “regplot” and “pROC” R packages. P < 0.05 was considered as statistically significant.

## Results

### Baseline characteristics of the patients

No statistically significant differences were observed in gender, age, FBG, Glycated hemoglobin, duration of diabetes mellitus, or complications between the groups (P > 0.05), as shown in Table 1.

### Comparison of glucose control

In the GM group, there was a lower incidence of hypoglycemia, severe hypoglycemia, and severe hyperglycemia compared to the control group. Additionally, the rate of glucose detection at 2 h postprandial was higher in the GM group than in the control group. The blood glucose attainment time was also significantly shorter in the GM group (P < 0.01), as shown in Table 1.

### Comparison of management metrics

The GM group had significantly fewer preoperative preparation days, hospital days, and lower health-care costs compared to the control group (P < 0.01 as shown in Table 1).

**Table 1:** Baseline characteristic of patients with control group or GM group

Characteristics	Level	Overall	Control group	GM group	P
n		4156	2458	1698	
Department (%)	Joint Surgery	324 (9.41)	144 (7.57)	180 (11.68)	< 0.0001
	Gastrointestinal Surgery	194 (5.63)	125 (6.57)	69 (4.48)	
	Gynecology	146 (4.24)	67 (3.52)	79 (5.13)	
	Hepatobiliary Surgery	326 (9.47)	142 (7.47)	184 (11.94)	
	Neurosurgery	224 (6.51)	141 (7.41)	83 (5.39)	
	Ophthalmology	607 (17.63)	483 (25.39)	124 (8.05)	
	Others	187 (5.43)	54 (2.84)	133 (8.63)	
	Otorhinolaryngology	220 (6.39)	94 (4.94)	126 (8.18)	
	Spine Surgery	368 (10.69)	151 (7.94)	217 (14.08)	
	Thoracic Surgery	301 (8.74)	159 (8.36)	142 (9.21)	
	Urological Surgery	546 (15.86)	342 (17.98)	204 (13.24)	
Sex (%)	Female	1924 (46.29)	1115 (45.36)	809 (47.64)	0.156
	Male	2232 (53.71)	1343 (54.64)	889 (52.36)	
Age (years)		60.993 (12.583)	61.278 (11.851)	60.582 (13.564)	0.08
Duration (years)		6.673 (5.916)	6.804 (5.939)	6.483 (5.880)	0.0853
Days_hospital (days)		9.830 (9.057)	10.396 (11.137)	9.011 (4.485)	< 0.0001
Preoperative preparation days		3.725 (3.689)	4.323 (4.418)	2.968 (2.262)	< 0.0001
Cost (RMB)		23262.166 (26748.962)	24096.775 (30802.414)	22053.997 (19383.044)	0.0155
Complications (%)	No	3414 (82.28)	2020 (82.38)	1394 (82.14)	0.8768
	Yes	735 (17.72)	432 (17.62)	303 (17.86)	
Glycated hemoglobin (%)		8.275 (2.086)	8.249 (2.190)	8.283 (2.055)	0.7836
FBG (mmol/L)		8.809 (3.603)	8.569 (3.593)	8.907 (3.603)	0.0602
Blood glucose attainment time (days)		3.316 (3.393)	3.938 (3.986)	2.416 (1.953)	< 0.0001
Grade 1 hypoglycemia (%)	No	3765 (90.59)	2130 (86.66)	1635 (96.29)	< 0.0001
	Yes	391 (9.41)	328 (13.34)	63 (3.71)	
Grade 2 hypoglycemia (%)	No	3837 (92.32)	2195 (89.30)	1642 (96.70)	< 0.0001
	Yes	319 (7.68)	263 (10.70)	56 (3.30)	
Grade 3 hypoglycemia (%)	No	4084 (98.27)	2393 (97.36)	1691 (99.59)	< 0.0001
	Yes	72 (1.73)	65 (2.64)	7 (0.41)	
Severe hyperglycemia (%)	No	3765 (90.59)	2256 (91.78)	1509 (88.87)	0.0019
	Yes	391 (9.41)	202 (8.22)	189 (11.13)	
2-hour postprandial glucose (%)	No	3909 (94.06)	2420 (98.45)	1489 (87.69)	< 0.0001
	Yes	247 (5.94)	38 (1.55)	209 (12.31)	

n, number of patients; Duration, the duration of diabetes; Days_hospital, hospital days; Cost, health-care costs; FBG, Fasting blood glucose; Unpaired t test (two sides), was used in two group measurement data. ANOVA test was used in in multi groups’ measurement data. Fisher’s exact test was used in enumeration data.

### Relationship of hospital days with multiple clinical characteristics

**Fig. 1:**
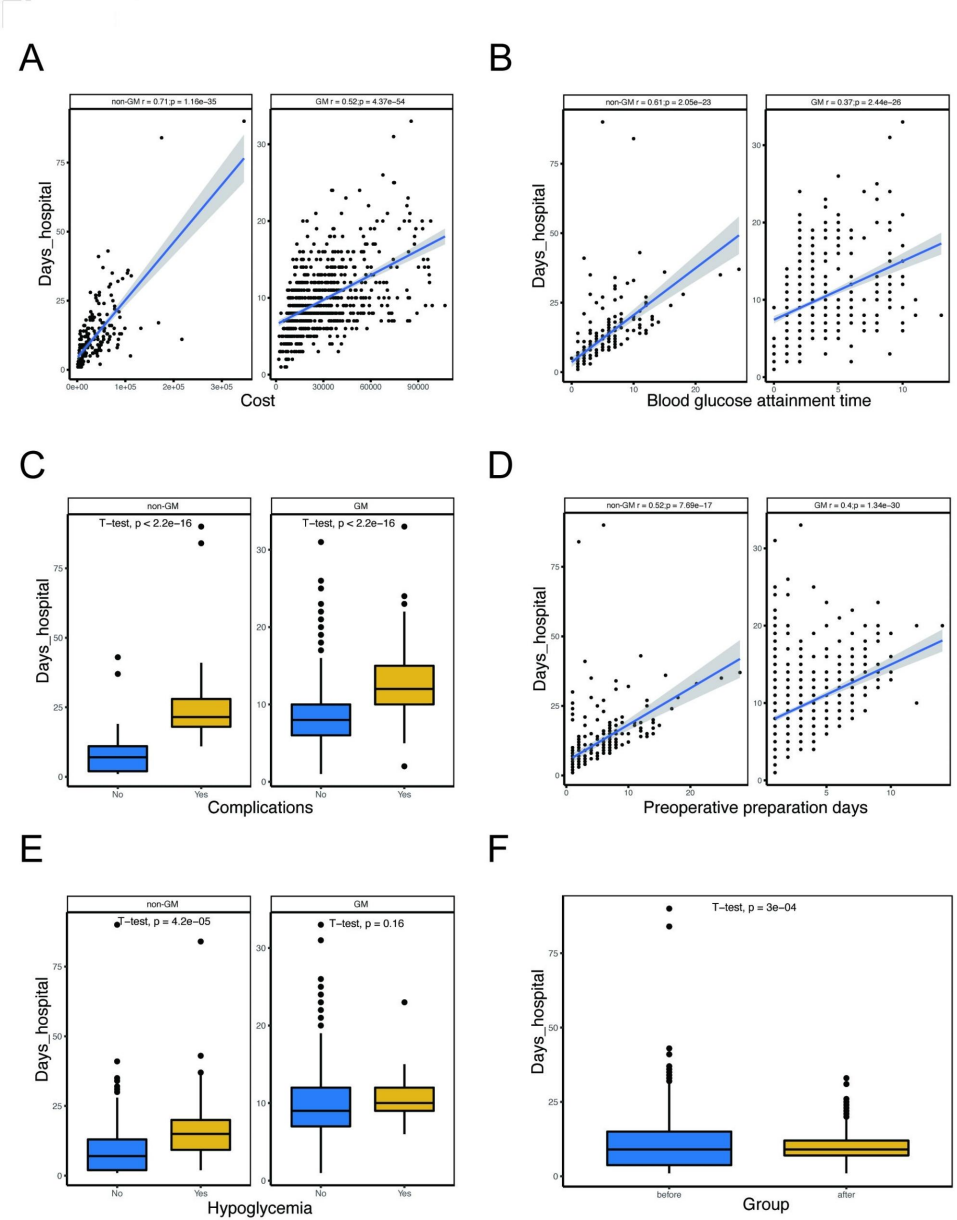
Relationship of hospital days with baseline characteristics. (**A**) Correlation analysis between hospital days and blood glucose attainment time. (**B**) Correlation analysis between hospital days and health-care costs. (**C**) Comparison of hospital days between patients without complications and patients with complications. (**D**) Correlation analysis between hospital days and preoperative preparation days. (**E**) Comparison of hospital days between patients without hypoglycemia and patients with hypoglycemia. (**F**) Comparison of hospital days between the control group and GM group

**Fig. 2:**
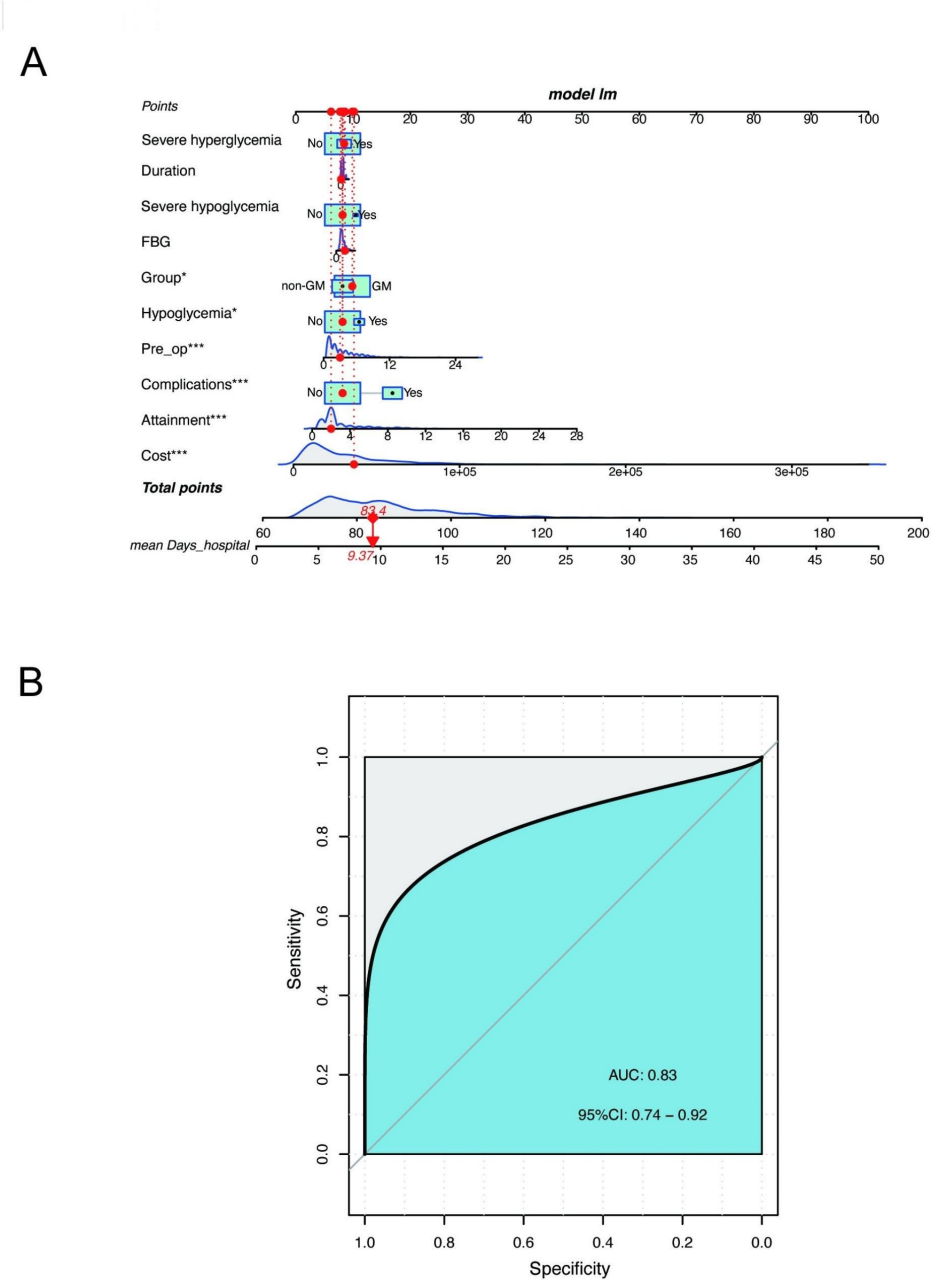
Establishment of the nomogram for predicting hospital days. (**A**) Nomogram with health-care costs, preoperative preparation days, blood glucose attainment time, complications, hypoglycemia incidence, and group for predicting hospital days among patients. Each level of predictor indicates a certain score. A total score was generated by a summary of the score of each predictor. *P < 0.05; **P < 0.01; ***P < 0.001. (**B**) ROC curve and AUC of the predictive model. ROC curve showed the accuracy of hospital days prediction on the basis of the nomogram

After observing the difference in hospital days between the GM and control groups (Fig. 1F), we conducted further analysis to examine the relationship between baseline characteristics and hospitalization days. We found that both the GM and control groups had positive associations between hospital cost, preoperative preparation days, and blood glucose attainment time with the number of hospitalization days (Fig. 1A, B, D). Additionally, the incidence of complications and hypoglycemia had a significant effect on the hospital days, with patients experiencing these conditions requiring longer hospitalization durations (Fig. 1C and E). To provide clinicians with an applicable tool for predicting hospital days, we constructed a nomogram that incorporated health-care costs, preoperative preparation days, blood glucose attainment time, complications, hypoglycemia incidence, and group (Fig. 2A). Receiver operating characteristic (ROC) curve analysis demonstrated that the nomogram had good accuracy for predicting hospital days (AUC 0.83, 95% CI 0.74–0.92) (Fig. 2B).

## Discussion

According to the Centers for Disease Control (CDC), diabetes has been declared a pandemic. In the United States, caring for individuals diagnosed with diabetes accounts for a quarter of healthcare costs, with more than half of those costs directly attributed to the disease [5]. In low-income countries, the total annual cost of diabetes treatment is $740.1, with direct costs of $646.7 and indirect costs of $93.65. These direct costs primarily include medications ($274.5) and hospitalization ($319.7) [17]. Patients with diabetes or hyperglycemia have longer hospital days, higher re-hospitalization rates, and greater morbidity and mortality compared to patients without these conditions [3, 6]. Poor glycemic control increases the risk of wound infections and complications, prolongs hospital days, and increases health-care costs. Additionally, hypoglycemia can be life-threatening for patients.

Traditionally, non-endocrinologists manage inpatient blood glucose levels independently under the direction of endocrinologists or guidelines [18]. However, the traditional blood glucose management model faces several problems, from personnel to equipment, that cannot meet the demand for further improving blood glucose management [19]. To address the issue, this study established an inpatient glucose team-based management and evaluated the glycemic control indicators, preoperative waiting days, hospital days, and health-care costs among surgical noncritical patients with diabetes. The results demonstrated a reduced incidence of hypoglycemia, severe hypoglycemia, and severe hyperglycemia in the GM group. The blood glucose attainment time was shortened, along with a decrease in preoperative preparation days, hospital days, and associated costs in the GM group. Additionally, the nomogram, including hospital cost, preoperative preparation days, blood glucose attainment time, complications, and hypoglycemia incidence, was established to create an applicable clinical evaluation instrument to predict hospital days among surgical noncritical patients with diabetes. The AUC [95% confidence interval] for the model was 0.83 [0.74–0.92], which indicated the good performance of the constructed prediction model. The implementation of inpatient glucose team-based management significantly improved glucose management for surgical patients with diabetes, resulting in better blood glucose levels and compliance rates. The inpatient glucose management model also led to reduced mean hospital days and health-care costs.

Insufficient mastery of glucose management among healthcare personnel in non-endocrine specialties and the lack of professional health education for hyperglycemic patients by non-endocrinology specialist nurses can negatively impact the quality of care and prolong hospitalization [20]. Hospitals often have low rates of endocrine consultation, hemoglobin A1C testing, and glucose treatment plans in discharge medical orders, with large gaps between correct treatment and guidelines after hypoglycemia. Some hospitals have implemented active blood glucose management for patients with diabetes in non-endocrine departments using traditional paper-based blood glucose data combined with a blood glucose management team, which has improved compliance rates and reduced hypoglycemia incidence [21]. However, this management mode still has limitations as it cannot view glucose data or adjust glucose-lowering plans in real time. Our hospital has addressed these issues by adopting an inpatient glucose management team consisting of endocrinologists, endocrinology nurses, non-endocrinology specialist liaison doctors/nurses, the nutrition department, the information department, and the pharmacy department. This multidisciplinary collaboration model efficiently manages blood glucose and reduces hospitalization days [22]. The advantages of inpatient blood glucose management include comprehensive and professional glucose management, improving compliance rates, shortening hospitalization time, simplifying the labor of non-endocrine healthcare workers, increasing work efficiency, and enhancing patient satisfaction, hospital visibility, and regional influence. Manual data copying errors are also avoided, and the Hitech system can make real-time adjustments to glucose-lowering plans.

Advances in glucose monitoring have improved clinical outcomes and quality of life for people with diabetes. The implementation of electronic tools as computerized physician order entry (CPOE) and clinical decision support systems (CDSS) in inpatient glycemic control and clinical benefits is expanding [23]. The study carried out by Toyoshima et al. [24] showed that in predominantly surgical patients the use of a digital tool had a low risk of hypoglycemia and a large number of blood glucose measurements within the therapeutic target. In our study, the GM group monitored blood glucose changes in real time through electronic tools thereby providing glycemic control for patients with diabetes in the surgical department. The results showed that the GM group had a significant increase in glucose detection rates at 2-hour postprandial glucose, a lower incidence of (severe) hypoglycemia and hyperglycemia compared to the control group. Interestingly, patients in the GM group had significantly fewer preoperative preparation days than those in the control group. Overall, these findings highlight the importance of effective glucose management and suggest that inpatient glucose management programs can be beneficial in achieving optimal outcomes. Maintaining proper blood glucose levels can also delay the progression of complications, minimize the incidence of postoperative infection, and accelerate wound healing. Another key benefit is that it involves physicians from different departments, enabling multidisciplinary collaboration and leading to more comprehensive treatment plans. Importantly, hospitalization factors such as cost, preoperative preparation days, blood glucose attainment time, complications, hypoglycemia incidence, and group were all found to be correlated with hospitalization days. Notably, patients in the GM group experienced significantly lower health-care costs and fewer hospital days. Overall, inpatient glucose team-based management saved consultation time for providers from different departments and improved efficiency while promoting positive patient collaboration. It also helped reduce hospital days and costs, highlighting the potential benefits of implementing a glycemic management program.

### Strength and limitations

Our study has several strengths. Firstly, it is based on a large sample size and highlights the significance of a comprehensive, multidisciplinary inpatient glucose team-based management model for patients with diabetes. Secondly, we identified factors influencing the hospital days for surgical patients with diabetes and visually represented the research results using a nomogram, providing a personalized prediction for the hospital days. The model also demonstrated relatively accurate predictive capabilities, as indicated by an AUC value of 0.83.

As a retrospective study, we also acknowledge some limitations in our research. Firstly, there may be unobserved and/or uncontrolled confounding factors that could impact the hospital days. Excluding patients with poorly controlled infections in this study may introduce selection bias. Therefore, further population-based prospective multicenter studies are required to better understand the factors influencing the hospital days in surgical patients with diabetes, specifically focusing on an inpatient glucose team-based management model.

## Conclusion

In this study, the implementation of an inpatient glucose team-based management approach significantly improved glycemic control and reduced hospital days among patients with diabetes in the surgical department. Multiple factors, including blood glucose attainment time, hypoglycemia incidence, preoperative preparation days, perioperative complications, and health-care costs, were found to influence the number of days spent in the hospital. These findings highlight the effectiveness of a team-based glucose management approach in optimizing glycemic control and improving healthcare resource utilization in surgical patients with diabetes. Future studies could further explore the long-term benefits and cost-effectiveness of this approach.

## Data Availability

The datasets used or analysed during the current study are available from the corresponding author on reasonable request.
